# Effect of EG00229 on Radiation Resistance of Lung Adenocarcinoma Cells

**DOI:** 10.7150/jca.56123

**Published:** 2021-08-24

**Authors:** Lele Cong, Junxuan Yi, Shuang Qiu, Rui Wang, Shunzi Jin, Rihua Jiang, Xianling Cong

**Affiliations:** 1 Department of Dermatology, China-Japan Union Hospital, Jilin University, Changchun 130033, Jilin, China.; 2 NHC Key Laboratory of Radiobiology, School of Public Health, Jilin University, Changchun, 130021, China.; 3 Tissue Bank, China-Japan Union Hospital, Jilin University, Changchun 130033, Jilin, China.

**Keywords:** Lung adenocarcinoma, non-small cell cancer, NRP1, radiation resistance, epithelial-mesenchymal transition (EMT), metastasis

## Abstract

**Background:** Neuropilin 1 (NRP1) is a pleiotropic receptor that interacts with multiple ligands and their receptors and plays a critical role in the process of tumor metastasis and radiation resistance in endothelial cells and tumor cells. In this study, we sought to investigate the mechanistic role of NRP1 in the radiation resistance of non-small cell lung cancer (NSCLC) cells and the role of EG00229 (an inhibitor of NRP1) on reversing radiation resistance.

**Materials and Methods:** A549 and H1299 NSCLC cells were used to construct radiation resistance models. Western blot, ELISA, and qRT-PCR were used to detect protein and mRNA levels of NRP1, epithelial-mesenchymal transition (EMT) markers, and molecules in signaling pathways. Immunofluorescence was used to measure changes in co-expression of NRP1 and VEGF-165 in radiation-resistant model cells. An immunoprecipitation assay was used to detect the binding capacity of NRP1 and VEGF-165.

**Results:** We successfully created two radiation resistant models (A549RR and H1299-RR). The expression levels of NRP1, EMT-related proteins, and proteins in metastasis-related pathways were increased in NSCLC cells with radiation resistance. After adding EG00229, the expression levels and binding capacity of NRP1 and VEGF-165 proteins were significantly reduced. The expression of EMT-related proteins and proteins in metastasis-related pathways were reduced in NSCLC cells with radiation resistance.

**Conclusion:** Our data provide an insight into the molecular mechanisms of radiation resistance and suggest that EG00229 may contribute to reversing the radiation resistance of NSCLC cells by inhibiting the binding of NRP1 and VEGF-165. Our findings could provide a novel theoretical and experimental foundation for improving the efficacy of lung cancer radiotherapy.

## Introduction

Lung cancer is the leading type of cancer and has the highest incidence and mortality, both worldwide and in China 1. Non-small cell lung cancer (NSCLC), a type of lung cancer that accounts for ~85% of all cases of lung cancer, and presents a severe threat to the health of the population 2. Surgery, radiotherapy, and chemotherapy are the main treatments for NSCLC 3. Radiotherapy is the primary treatment for early stage NSCLC patients who are considered inoperable 4. Radiation resistance is a major cause of therapeutic failure in NSCLC, leading to tumor recurrence and metastasis 5. However, the molecular mechanisms of intrinsic resistance to radiotherapy in NSCLC are not clear.

Neuropilins (NRPs) are cell surface glycoproteins that mediate neuronal guidance and angiogenesis 6. NRP1 is one of two NRP genes and contributes to tumor angiogenesis and tumor malignancy 7. Vascular endothelial growth factor (VEGF) is a key regulator of angiogenesis and increases the survival, proliferation, and migration of endothelial cells 8-10. VEGF165 is one of the most common subtypes of VEGF and binds to VEGFR2 and NRP1 11. Studies have shown that NRP1 can enhance the resistance of tumor cells to radiation through the activity of VEGF and semaphorin, among others 12, 13. EG00229, a small molecule inhibitor, is an antagonist of the NRP1 receptor and can inhibit the binding of VEGF165 to the NRP1 b1 domain 14. Thus, EG00229 may reverse the radiation resistance of lung adenocarcinoma cells by inhibiting the binding of NRP1 and VEGF and influencing the expression of NRP1 and VEGF.

The aims of this study were to investigate the molecular mechanisms underlying resistance to radiation in NSCLC cells and to explore the inhibitory role of EG00229 on radiation resistance.

## Materials and Methods

### Patients and specimens

Surgical specimens of cancer tissue and paired adjacent normal tissue were collected from 5 patients with lung adenocarcinoma who underwent surgery at China-Japan Union Hospital of Jilin University from 2019 to 2020. No patients received chemotherapy and radiotherapy before surgery. All tissue samples were immediately frozen in liquid nitrogen and store at -80 °C in tissue bank of the hospital. This study was approved by the Ethics Committee of the China-Japan Union Hospital of Jilin University, and all participants provided written informed consent.

### Cell lines and culture

The A549 and H1299 human lung adenocarcinoma cell lines were obtained from the Type Culture Collection of the Chinese Academy of Sciences (Shanghai, China). A549 and H1299 cell lines were cultured in DMEM (Gibco) and RPMI-1640 medium (Gibco, Grand Island, NY, USA), respectively, supplemented with 10% fetal bovine serum (HyClone, Waltham, USA) and 1% penicillin-streptomycin at 37 °C in a humidified atmosphere of 5% CO2.

### Reagents

Lyophilized EG00229 powder (MedChemExpress, NJ, USA) was dissolved in sterile dimethyl sulfoxide (DMSO) after centrifugation at 12,000 rpm for 10 min.

### Cell proliferation assay

Cell proliferation was measured using the Cell Counting Kit-8 (CCK-8) (MedChemExpress, NJ, USA), according to the manufacturer's instructions. Cells were seeded in a 96-well plate and cultured in a 37 °C incubator for 24 h. After adherence they were irradiated with X-rays. We added 10 μl of CCK-8 reagent to the test well. The absorbance was measured at 450 nm using an Epoch BioTek® ELX 800 plate reader (BioTek, Winooski, VT).

### Immunohistochemical staining

Immunohistochemistry (IHC) staining was performed with horseradish peroxidase (HRP) conjugates using DAB detection. 4 µm thick sections were cut from paraffin-embedded blocks, 0.01 mol/l citrate buffer (pH 6.0) and 3% hydrogen peroxide were used for antigen retrieval and endogenous peroxidase activity blocking. The sections were incubated with the primary antibody (NRP1: 1:300, Abcam, Cambridge, MA, USA; VEGF-165:1:400, Biosson, Beijing, China) at 4°C overnight. The sections were following incubated with the corresponding secondary antibody at room temperature, then they were finally examined by light microscopy. Each slide was scored in a blinded fashion by two pathologists according to the manufacturer's recommended criteria. The immunostaining was read in a semiquantitative manner and graded as follows: 0, negative; 1, weak; 2, moderate; 3, strong. The frequency of positive cells was defined as: 0, less than 5%; 1, 5% to 25%; 2, 26% to 50%; 3, 51% to 75%; and 4, greater than 75%. The final score was defined the product of the intensity and frequency. Scores of 0 to 7 were considered low expression and scores of 8 to 12 were considered high expression.

### Immunoprecipitation

After cells were cultured with EG00229 for 10 h, the cell pellet was collected. An appropriate amount of cell lysis buffer (containing protease inhibitor) was added to the cell pellet. The supernatant was obtained after centrifugation and 10% of the supernatant was extracted for western blot analysis. We then added 1 μg of the corresponding antibody to the supernatant of the remaining lysate and incubated overnight. After incubation, pretreated protein A agarose was added to the lysate to couple with the antibody and form an immunoprecipitation reaction.

### Clone formation assay

After adherence to the well, the cells were irradiated with 2, 4, 6, or 8 Gy of radiation and cultured in a 37 °C incubator for 14 days. Cells were then fixed with 1 ml of 4% paraformaldehyde for 20 min followed by staining with 1 ml crystal violet for 30 min. The colony formation rate was calculated using the following formulas:

SF = 1 - (1-e^-D/D0^)^N^;

Dq=D0 log(N)

where SF is the survival fraction, D is the radiation dose (Gy), D0 is the average lethal dose, Dq is the quasi-field dose, and N is the extrapolation number.

### RNA extraction and quantitative real-time PCR

Total RNA was isolated from cells with TRIzol (Invitrogen, Carlsbad, CA, USA), according to the manufacturer's protocol. Reverse transcription was performed using PrimeScript qRT-PCR kit (TaKaRa, Dalian, China). cDNA was amplified by SYBR Green assay (TaKaRa), as per the manufacturer's instructions, with the CFX96Touch Real-Time PCR System (Applied Biosystems, Foster City, CA, USA) and using GAPDH as an internal control. The qRT-PCR primers used are listed in Supplementary Table 1. All reactions were performed in triplicate in separate tubes to permit quantification of target regions. The relative quantification of gene expression was determined as 2^-∆∆Ct^ 15, 16.

### Protein extraction and western blot analysis

Cells were lysed with ice-cold radioimmunoprecipitation assay buffer for 30 min (RIPA, Beyotime Biotechnology, Shanghai, China). The protein concentration was measured by BCA assay (Beyotime Biotechnology, Shanghai, China). Protein lysates were then subjected to sodium dodecyl sulfate-polyacrylamide gel electrophoresis (Millipore, Billerica, MA, USA), transferred onto nitrocellulose membranes, and immunoblotted with primary antibodies, followed by the matched secondary antibodies. Primary antibodies used in this study were anti-α-SMA (1:1000; Bioword Technology, MN, USA), anti-Vimentin (1:1000; Bioword Technology, MN, USA), anti-β-catenin (1:1000; Bioword Technology, MN, USA), anti-PI3K (1:1000; Bioword Technology, MN, USA), anti-STAT3 (1:1000; Bioword Technology, MN, USA), anti-VEGF-165 (1:1000; Biosson, Beijing, China), anti-Akt (1:1000; Cell Signaling Technology, Shanghai, China), anti-*p*-Akt (1:1000; Cell Signaling Technology), anti-mTOR (1:1000; Cell Signaling Technology), anti-*p*-mTOR (1:1000; Cell Signaling Technology), anti-CXCR4 (1:1000; Bioword Technology, MN, USA), anti-N-cadherin (1:1000; Bioword Technology, MN, USA), anti-NRP1 (1:1000; Abcam, Cambridge, MA, USA), and anti-GAPDH (1:5000; Proteintech Group Inc., Chicago, IL, USA).

### Enzyme-linked immunosorbent assay

An enzyme-linked immunosorbent assay (ELISA) was used to quantify the concentration of IL-6 and SDF-1 in each group. ELISA kits for the detection of each cytokine were obtained from R&D Systems (Minneapolis, MN, USA). The assays were performed in duplicate with 50 µl of sample added to each well, as per the manufacturer's instructions. The readings were taken using an Epoch BioTek® ELX 800 plate reader (BioTek, Winooski, VT, USA). The OD was read at 450 nm with reference set to 630 nm. A standard curve was prepared for each cytokine, and the corresponding curve formulas were used to calculate the concentration of each sample.

### Immunofluorescence

Cells were rinsed 3 times in phosphate buffered saline (PBS), fixed with 4% paraformaldehyde for 15 min, and then incubated with 0.5% Triton X-100 (Thermo Fischer Scientific, San Jose, CA) for 20 min. After blocking in a 5% solution of bovine serum albumin (Sigma, San Antonio, USA) for 30 min at 37 °C, cells were incubated with primary antibodies against NRP1, VEGF-165, or F-actin at 4 °C overnight, followed by Cy3-labelled anti-rabbit IgG secondary antibody for 1 h. The cells were then visualized using a fluorescent microscope.

### Wound healing assay

A wound-healing assay was used to assess migration ability. When cells reached 90% confluence, a 200 μl pipette tip was used to make a straight scratch. The cells were then rinsed with PBS, cultured in DMEM medium without serum for 12 h, and cultured in DMEM medium with serum for 12 h. Images were captured by a microscope to determine the width of the wounded area.

### Irradiation protocol

A549 and H1299 cells were sham-irradiated or exposed to ionizing radiation (IR) at 30 Gy (dose rate: 1.02 Gy/min and 0.75 Gy/min, respectively; source skin distance: 70 cm and 60 cm, respectively; voltage: 180 kV and 320 kV, respectively; current: 20.0 mA and 12.5 mA, respectively) by an X-ray generator (Model X-RAD320iX; Precision X-Ray, Inc., North Branford, CT, USA). The cells were then exposed to 6 Gy X-ray irradiation after cell adherence and cultured for 10-12 days. When the growth of the cells had reached 80% confluence, the cells were irradiated with 6 Gy X-rays. The entire process was repeated 5 times for a total radiation dose of 30 Gy. After the last irradiation, the formed clonal cells were radiation-resistant. The radiation-resistant cell models of A549 and H1299 cells were named A549-RR and H1299-RR, respectively.

### Statistical analysis

Data are presented as the mean ± standard deviation (SD). Comparisons between groups were analyzed using Student's t-test. All statistical analyses were performed using IBM SPSS version 24.0 (SPSS, Inc., Chicago, USA). *P*-values <0.05 were considered to indicate statistical significance.

## Results

### Expression of NRP1 and VEGF-165 in lung adenocarcinoma tissues

**Figure 1:**
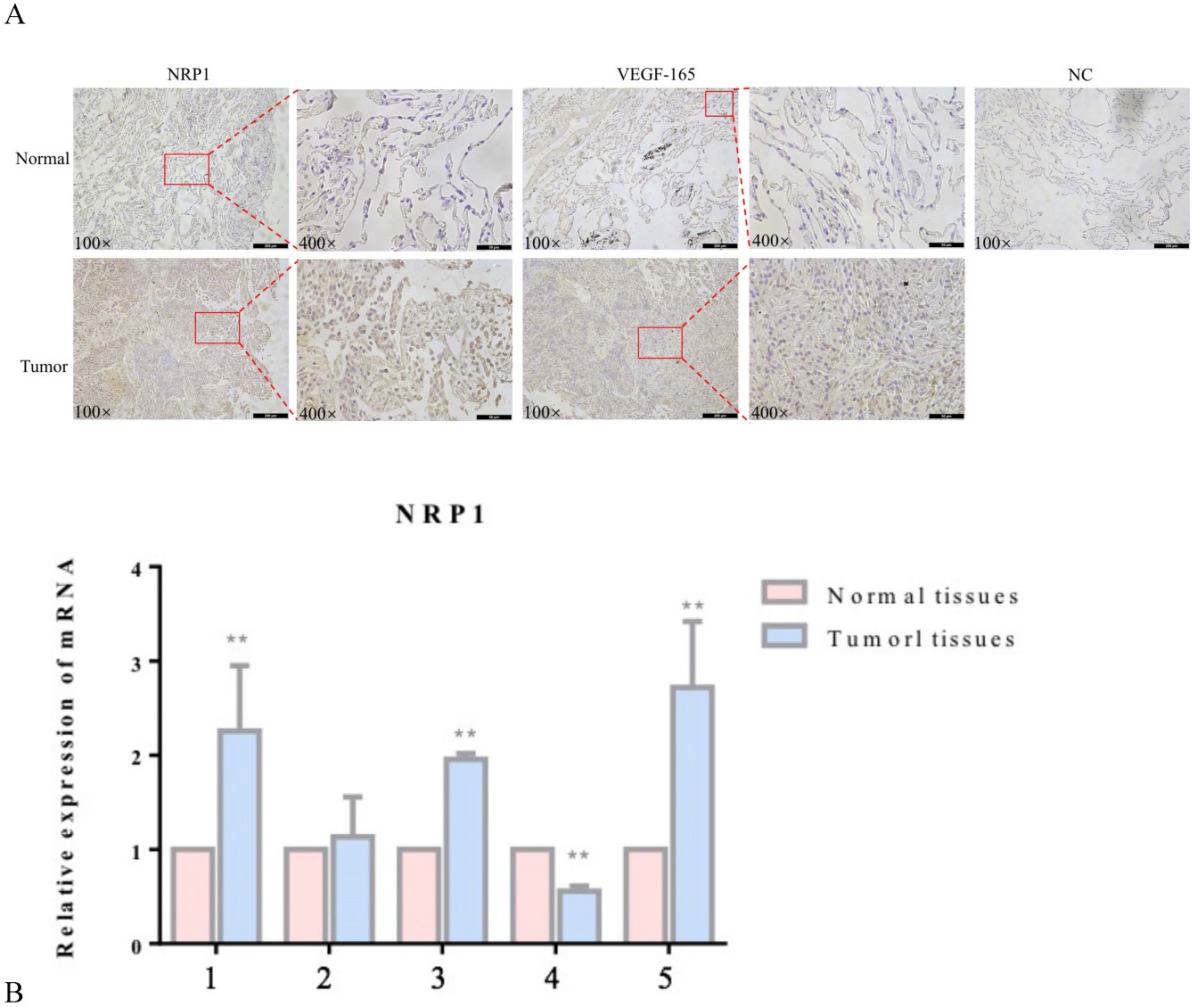
** Expression of NRP1 and VEGF-165 in lung adenocarcinoma.** Representative IHC stains of NRP1 and VEGF-165 in tumor tissues and adjacent normal tissues (**A**) qRT-PCR of NRP1 expression in 5 pairs of lung adenocarcinoma tissues and adjacent normal tissues (**B**). **P <0.01.

NRP1 and VEGF-165 expression in tumor tissues and adjacent healthy tissues of 5 patients with lung adenocarcinoma was detected by immunohistochemistry (IHC) and qRT‐PCR. Compared with adjacent healthy tissues, the expression levels of NRP1 and VEGF-165 were significantly increased in tumor tissues (P<0.05; Figure 1).

### Construction and identification of the cell model

**Figure 2:**
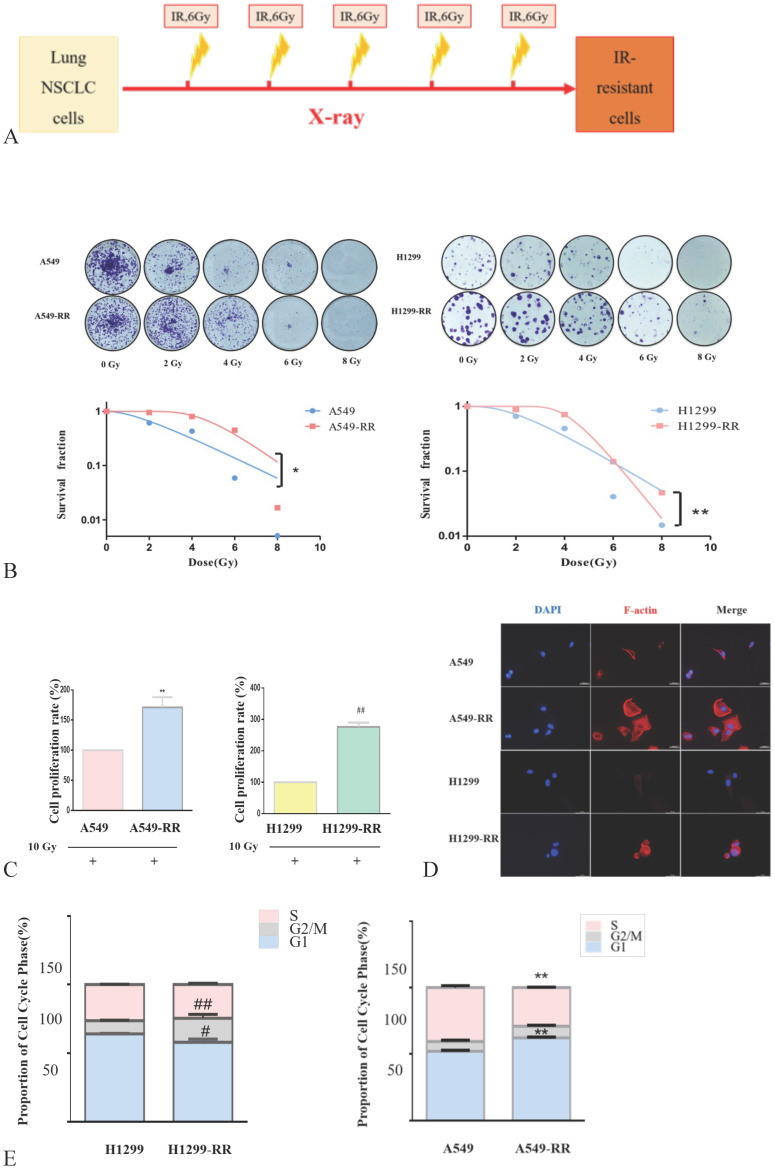

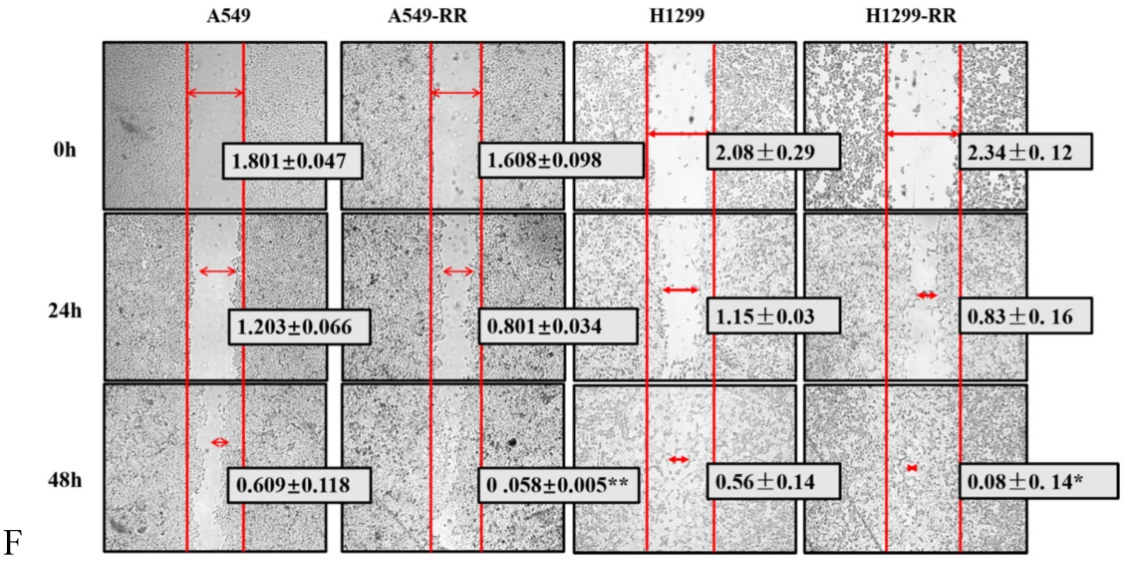
** Construction and identification of the radiation resistant lung adenocarcinoma cells.** Strategy for the generation of A549-RR and H1299-RR cells (**A**). Effect of X-ray irradiation on colony forming ability (**B**) and proliferative ability (**C**). Morphology changes in A549-RR and H1299-RR cells (**D**). Changes in cell cycle progression (**E**). Wound-healing assay of A549-RR and H1299-RR cells (**F**). A549-RR cells compared with the A549 cells **P <0.01; H1299-RR cells compared with the H1299 cells ##*P* <0.01 (n = 3).

The strategy for the generation of A549-RR and H1299-RR cells is presented in Figure 2A. The colony formation of cells decreased significantly as the radiation dose increased. A549-RR and H1299-RR cells had stronger colony forming ability than A549 and H1299 cells after irradiation with the same dose (Figure 2B). The survival curve-fitting results of single click multi-target models showed that the SF of A549, A549-RR, H1299 and H1299-RR cells was 0.612, 0.952, 0.706, and 0.893, respectively, (Table 1) with 2 Gy irradiation. After irradiation, the D0 value of A549 and H1299 cells decreased and the DQ value increased. This indicated that the radiosensitivity of A549 and H1299 cells decreased, and the ability of cells to accumulate sublethal damage was enhanced. The radiation resistance of A549-RR and H1299-RR cells was higher than that of A549 and H1299 cells.

In the process of constructing the radiation resistance cell models (A549-RR and H1299-RR), CCK-8 test results showed that after 24 h with 10 Gy X-ray irradiation, the proliferative ability of the radiation-resistant cell models (A549-RR and H1299-RR) was stronger than that of A549 and H1299 cells (*P* < 0.01, Figure 2C).

The cell volume of A549-RR and H1299-RR cells increased, and the cytoplasm appeared more spindle-shaped. Immunofluorescence staining showed that A549-RR and H1299-RR cells had significant F-actin stress fiber networks in the membrane and perinuclear regions. A549 cells only displayed short, dispersed, and non-organized fiber networks in the membrane region while H1299 cells did not display any obvious fiber networks in the membrane region. These results suggested that the enhancement of radiation resistance could affect the morphology of cells (Figure 2D,E).

Flow cytometry results showed that, compared with A549 cells, A549-RR cells displayed G1 phase arrest and S phase shortening. Compared with H1299 cells, H1299-RR cells showed G2/M phase arrest and G1 phase shortening. These findings indicated that these two types of radiation-resistant lung adenocarcinoma cells could affect cell cycle progression through different cell cycle distributions (Figure 2F).

The results of the wound-healing assay showed that the migration distance of A549-RR cells increased significantly compared with that of A549 cells 48 h after the scratch was established (*P* < 0.01). The migration distance of H1299-RR cells was also increased compared with that observed in H1299 cells (*P* < 0.05), suggesting that increased radiation resistance of cells was associated with a stronger migratory ability (Figure 2G). These results showed that the radiation resistance models (A549RR and H12 99-RR) were successfully established.

### Expression of NRP1 and EMT-related markers in radiation resistance models

**Figure 3:**
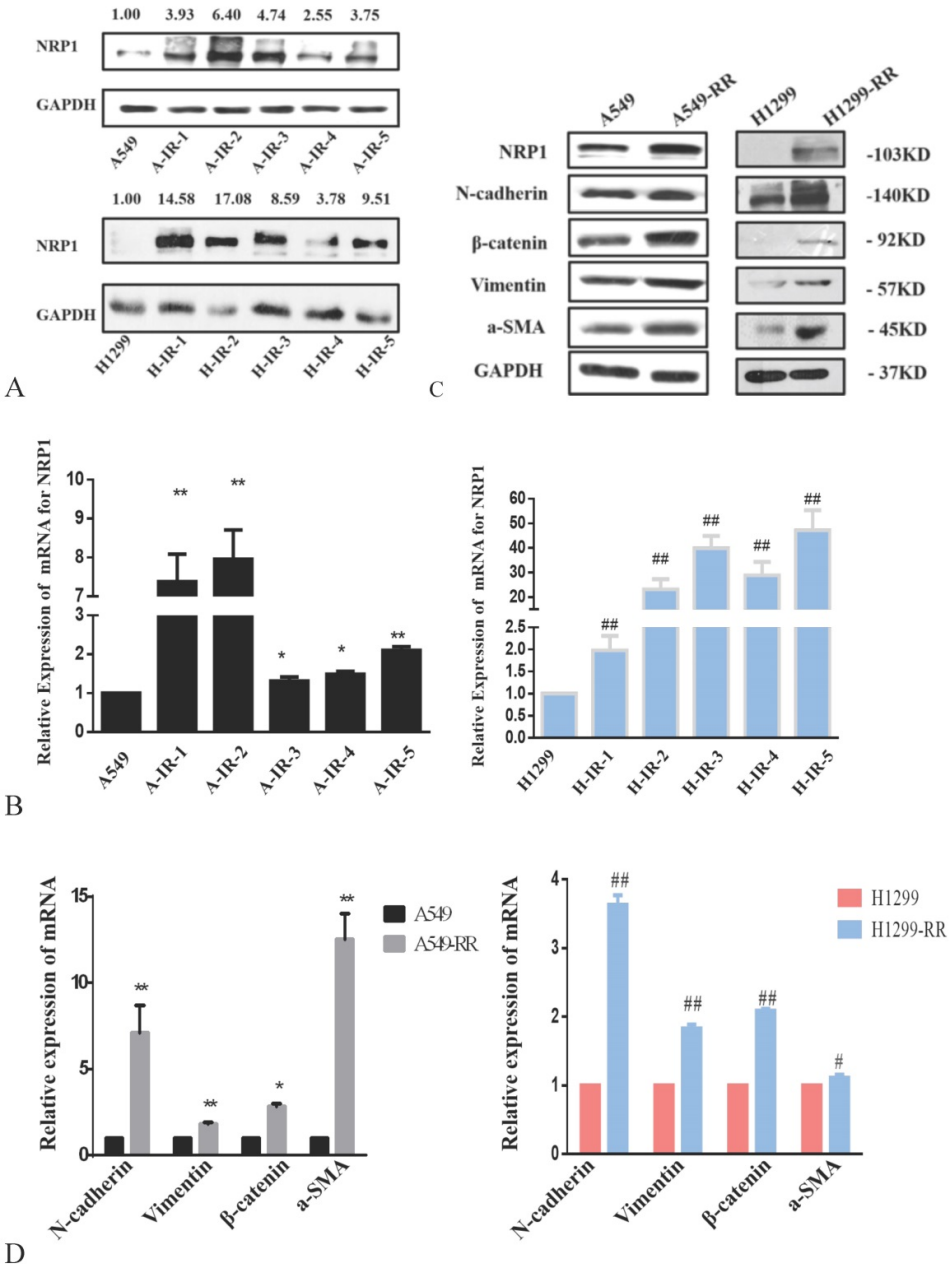
** Analysis of NRP1 and EMT-associated protein expression in radiation resistant and parental cells.** Changes in NRP1 expression during construction of the A549RR and H1299-RR models at the protein (**A**) and mRNA level (**B**). Changes in NRP1 and EMT-associated protein expression in radiation resistant and parental cells at the protein (**C**) and mRNA level (**D**). A549-RR cells compared with the A549 cells. *P <0.05, **P <0.01; H1299-RR cells compared with the H1299 cells ##*P* <0.01 (n=3).

The protein expression level of NRP1 peaked after the second irradiation and tended to be stable after the fifth irradiation in both A549RR and H1299-RR cells (Figure 3A). After the fifth irradiation, the mRNA expression level of NRP1 was approximately 2.10 times higher in A549RR cells than in A549 cells (*P* < 0.01, Figure 3B) and 47.16 times higher in H1299-RR cells than in H1299 cells (*P* < 0.01, Figure 3B).

Western blot analysis of EMT-related protein markers revealed that the expression levels of N-cadherin, β-catenin, vimentin, and α-SMA were significantly increased in A549-RR cells compared to A549 cells (Figure 3C). The mRNA expression levels of EMT markers in cells were detected by qRT-PCR. Compared with A549 cells, N-adherin was 7.10 times higher (*P* < 0.01), β-catenin was 2.85 times higher (*P* < 0.05), vimentin was 1.82 times higher (*P* < 0.01), and α-SMA was 12.53 times higher (*P* < 0.01) in A549-RR cells (Figure 3D). In H1299-RR cells, the expression levels of EMT-related protein markers (N-cadherin, β-catenin, vimentin, and α-SMA) were also higher than those in H1299 cells (Figure 3C). Compared with H1299 cells, the mRNA expression level of N-adherin was 3.63 times higher (*P* < 0.01), β-catenin was 2.09 times higher (*P* < 0.01), vimentin was 1.83 times higher (*P* < 0.01), and α-SMA was 1.11 times higher in H1299-RR cells (*P* < 0.05) (Figure 3D).

### Mechanism of cell metastasis related pathways after radiation resistance in lung adenocarcinoma cells

**Figure 4:**
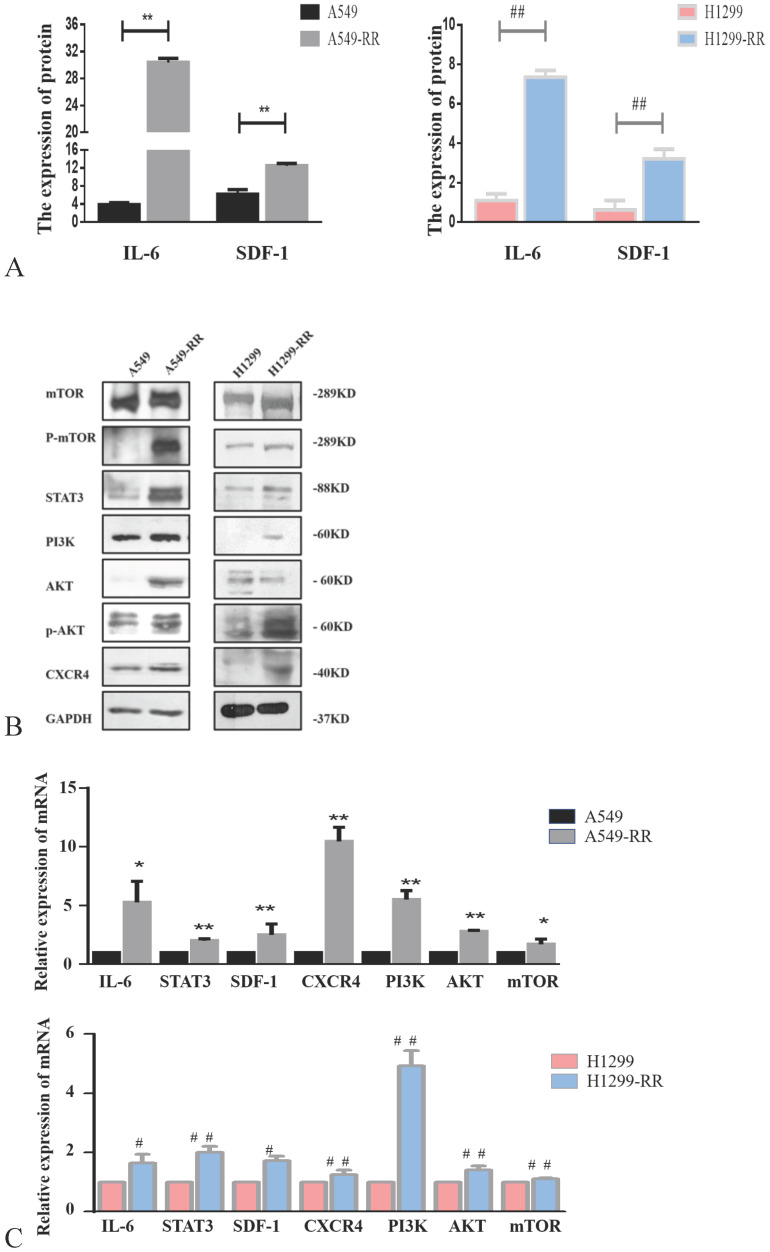
** Analysis of the expression of proteins in the IL-6/STAT3, SDF-1, and PI3K/AKT/mTOR pathways in radiation resistant and parental cells.** Changes in secretion of IL-6 and SDF-1 in A549-RR and H1299-RR cells (**A**). Changes in mTOR, P-mTOR, STAT3, PI3K, AKT, p-AKT, and CXCR4 in A549-RR and H1299-RR cells at the protein level (**B**). Changes in IL-6, STAT3, SDF-1, CXCR4, PI3K, AKT, and mTOR in A549-RR and H1299-RR cells at the mRNA level (**C**). A549-RR cells compared with the A549 cells *P <0.05, **P <0.01; H1299-RR cells compared with the H1299 cells #P <0.05, ##P <0.01 (n=3).

Western blot and ELISA were used to detect the expression levels of proteins in metastasis-related pathways (IL-6/STAT3, SDF-1/CXCR4 and PI3K/AKT/mTOR). The expression levels of IL-6, STAT3, SDF-1, CXCR4, PI3K, AKT, p-AKT, mTOR and p-mTOR proteins were all significantly increased in A549-RR cells compared to A549 cells (Figure 4A,B). The mRNA expression levels of these markers were detected by qRT-PCR. Compared with A549 cells, IL-6 was 5.3 times higher (*P* < 0.05), STAT3 was 2.0 times higher (*P* < 0.01), SDF-1 was 2.5 times higher (*P* < 0.01), CXCR4 was 10.5 times higher (*P* < 0.01), PI3K was 5.5 times higher (*P* < 0.01), AKT was 2.8 times higher (*P* < 0.01), and mTOR was 1.7 times higher (*P* < 0.05) in A549-RR cells (Figure 4C). In H1299-RR cells, the protein expression levels of IL-6, STAT3, SDF-1, CXCR4, PI3K, p-AKT, and p-mTOR were also higher than the levels observed in H1299 cells; however, this did not reach the level of statistical significance for AKT and mTOR (Figure 4A,B). Compared with H1299 cells, the mRNA expression level of IL-6 was 1.6 times higher (*P* < 0.05), STAT3 was 2.0 times higher (*P* < 0.01), SDF-1 was 1.7 times higher (*P* < 0.05), CXCR4 was 1.3 times higher (*P* < 0.01), PI3K was 4.9 times higher (*P* < 0.01), AKT was 1.4 times higher (*P* < 0.01), and mTOR was 1.1 times higher (*P* < 0.01) in H1299-RR cells (Figure 4C).

### Mechanism of EG00229 in lung adenocarcinoma cells with radiation resistance

**Figure 5:**
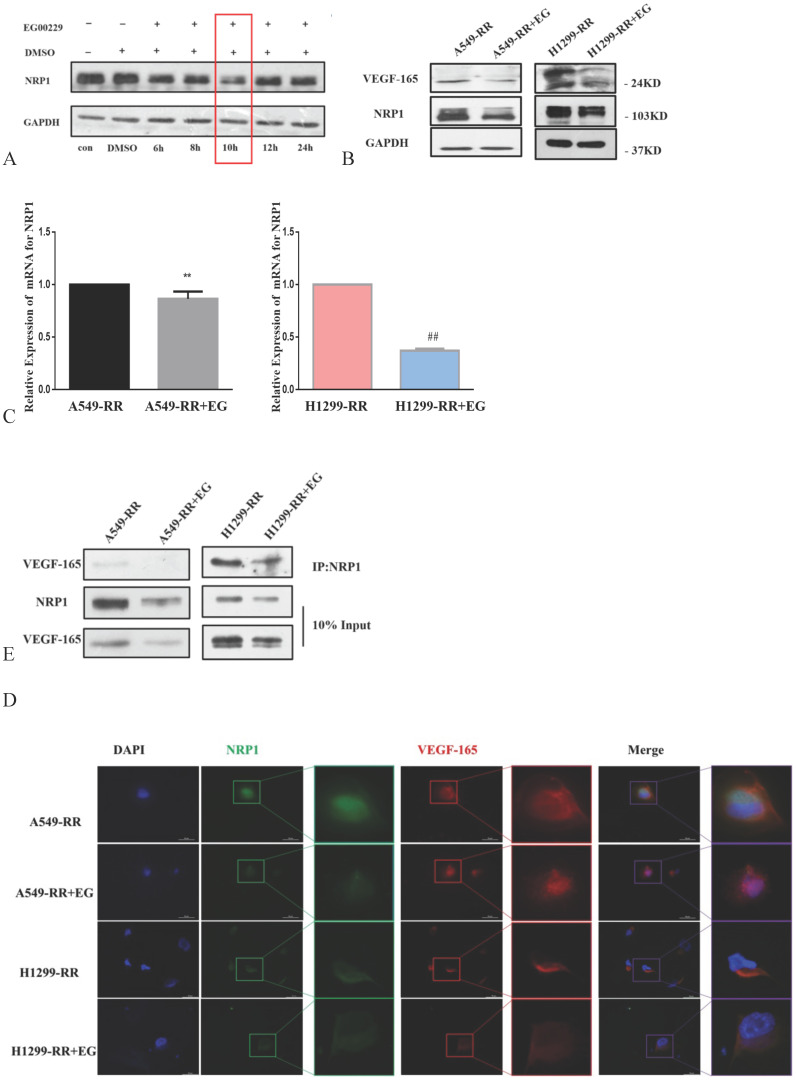
** Effect of EG00229 on NRP1 and VEGF-165 in two radiation-resistant cell models.** Changes in the expression of NRP1 following incubation with EG00229 (**A**). The effect of inhibitors on NRP1 and VEGF-165 in radiation-resistant model cells at the protein (**B, D**) and mRNA level (**C**). The effects of EG00229 on the binding capacity of NRP1 and VEGF-165 proteins (**E**). A549-RR+EG (EG is EG00229) cells compared with the A549-RR cells **P <0.01; H1299-RR+EG cells compared with the H1299-RR cells ##*P* <0.01 (n = 3).

The expression level of NRP1 reached its nadir following the addition of EG00229 for 10h (Figure 5A). The expression levels of NRP1 and VEGF-165 proteins were downregulated in both A549-RR and H1299-RR cells after adding EG00229 for 10h (Figure 5B). Similarly, the mRNA expression level of NRP1 decreased by 0.82 in A549-RR cells (*P* < 0.05) and by 0.37 times in H1299-RR cells (*P* < 0.01) after adding EG00229 for 10h (Figure 5C). Immunofluorescence analysis showed that the co-expression of NRP1 and VEGF-165 in A549-RR and H1299-RR cells had also decreased after adding EG00229 for 10 h (Figure 5D), while protein immunoprecipitation analysis showed that the binding capacity of NRP1 and VEGF-165 proteins was significantly reduced in A549-RR and H1299-RR cells after adding EG00229 for 10 h (Figure 5E).

**Figure 6:**
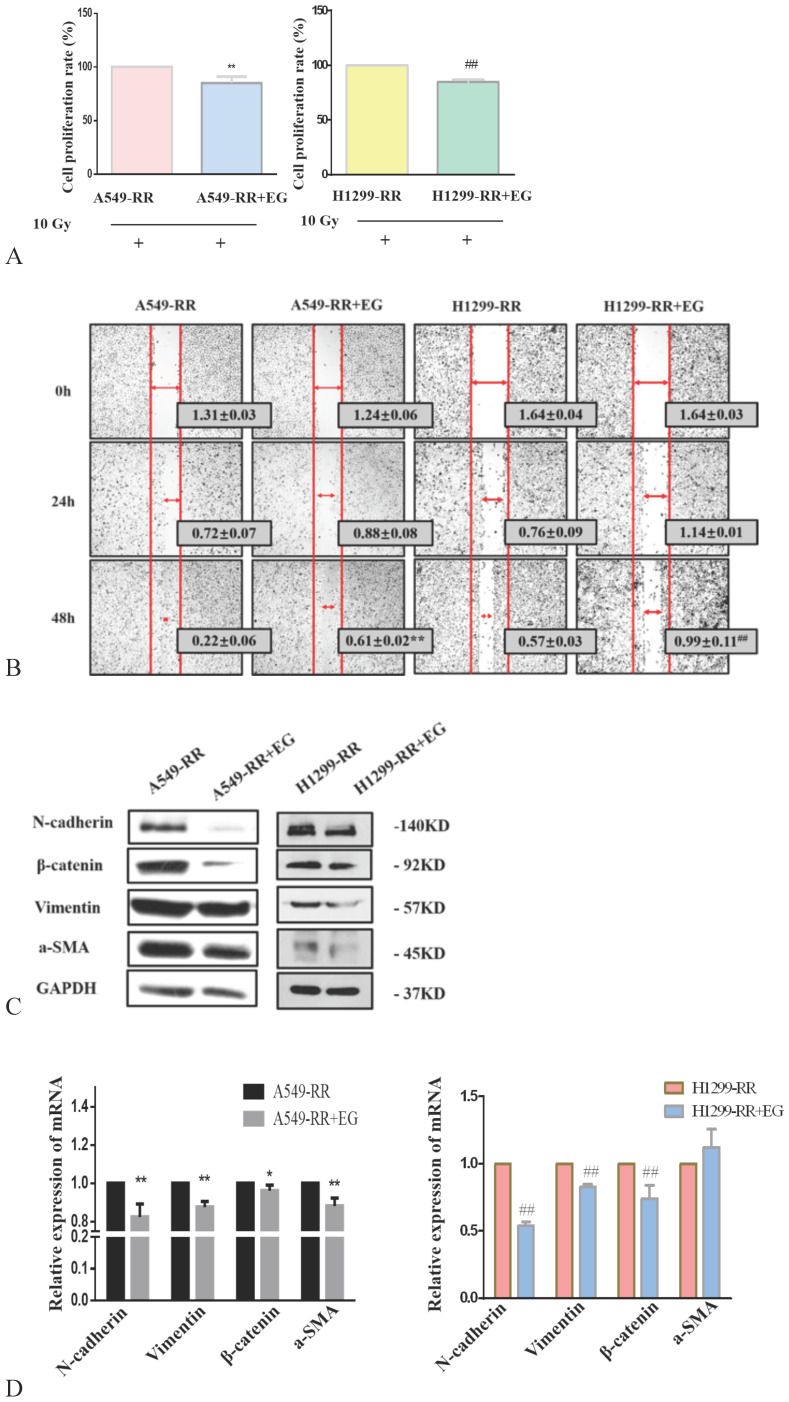
** Effect of EG00229 on EMT-associated protein expression in two radiation resistant cell models.** Effect on proliferative ability of A549-RR and H1299-RR cells (**A**). Wound-healing assay of A549-RR and H1299-RR cells after addition of EG00229 (**B**). The effect of EG00229 on the expression of EMT-associated proteins in radiation resistant cells at the protein (**C**) and mRNA level (**D**). A549-RR+EG (EG is EG00229) cells compared with the A549-RR cells *P <0.05, **P <0.01; H1299-RR+EG cells compared with the H1299-RR cells #*P* <0.05, ##*P* <0.01 (n=3).

After 10 Gy X-ray irradiation for 24 h and the addition of EG00229 for 10 h, the proliferative capacity of A549-RR and H1299-RR cells decreased (all *P* < 0.01) (Figure 6A). The results of the wound-healing assay showed that the migration distances of A549-RR and H1299-RR cells were significantly reduced 48 h after the scratch was established and 10h after adding EG00229 (all *P* < 0.01, Figure 6B). These findings suggested that the radiation resistance and migratory ability had decreased. Western blot analysis of EMT-related protein markers revealed that the expression levels of N-cadherin, β-catenin, vimentin, and α-SMA proteins were significantly decreased in A549-RR and H1299-RR cells after adding EG00229 for 10 h (Figure 6C). Specifically, N-adherin was 0.8 times lower (*P* < 0.01), β-catenin was 0.92 times lower (*P* < 0.05), vimentin was 0.82 times lower (*P* < 0.01), and α-SMA was 0.88 times lower (*P* < 0.01) in A549-RR cells (Figure 6D). In H1299-RR cells, N-adherin expression was 0.54 times lower (*P* < 0.01), β-catenin was 0.74 times lower (*P* < 0.01), and vimentin was 0.83 times lower (*P* < 0.01) after adding EG00229 for 10 h (Figure 6D).

**Figure 7:**
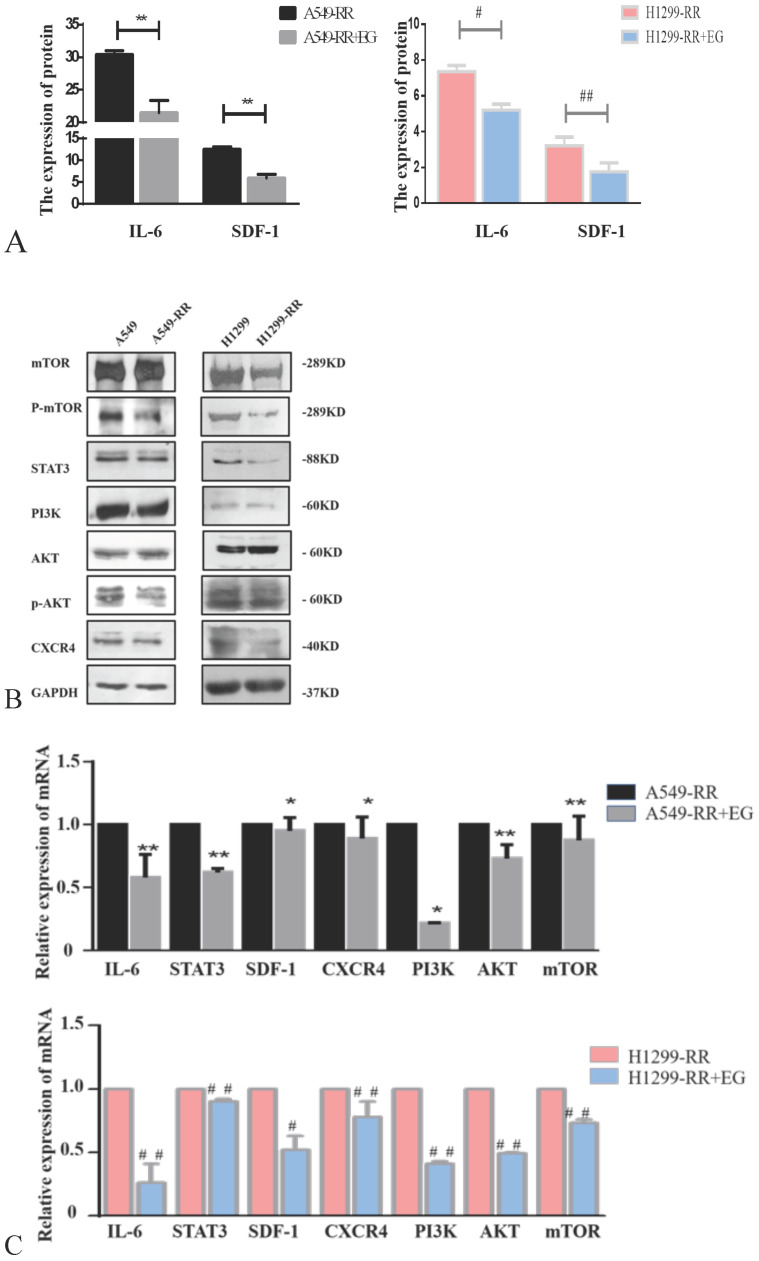
** Effect of EG00229 on IL-6/STAT3, SDF-1, and PI3K/AKT/mTOR pathways in two radiation resistant cell models.** The effect of EG00229 on the secretion of IL-6 and SDF-1 in A549-RR and H1299-RR cells (**A**). The effect of EG00229 on mTOR, P-mTOR, STAT3, PI3K, AKT, p-AKT, and CXCR4 in A549-RR and H1299-RR cells at the protein level (**B**). The effect of EG00229 on IL-6, STAT3, SDF-1, CXCR4, PI3K, AKT, and mTOR on A549-RR and H1299-RR cells at the mRNA level (**C**). A549-RR+EG (EG is EG00229) cells compared with the A549-RR cells *P <0.05, **P <0.01; H1299-RR+EG cells compared with the H1299-RR cells #*P* <0.05, ##*P* <0.01 (n=3).

Western blot and ELISA detection of the proteins in the IL-6/STAT3 pathway, which is related to cell proliferation, revealed that the expression levels of IL-6 and STAT3 proteins were all significantly decreased in A549-RR cells after adding EG00229 for 10 h (Figure 7A,B). The mRNA expression levels of IL-6 and STAT3 in A549-RR cells were detected by qRT-PCR. IL-6 was 0.58 times lower (*P* < 0.01) and STAT3 was 0.62 times lower (*P* < 0.01) in A549-RR cells after adding EG00229 for 10 h (Figure 7C). In H1299-RR cells, the protein expression levels of IL-6 and STAT3 were also reduced (Figure 7A,B). The mRNA expression levels of IL-6 and STAT3 were 0.26 times (*P* < 0.01) and 0.90 times (*P* < 0.01) lower, respectively, after adding EG00229 for 10 h (Figure 7C).

The expression levels of SDF-1 and CXCR4 proteins were all significantly decreased in A549-RR cells after adding EG00229 for 10 h (Figure 7A,B). SDF-1 was 0.95 times lower (*P* < 0.05) and CXCR4 was 0.89 times lower (*P* < 0.05) in A549-RR cells after adding EG00229 for 10 h (Figure 7C). In H1299-RR cells, the protein expression levels of SDF-1 and CXCR4 had also decreased (Figure 7A,B). The mRNA expression levels of SDF-1 and CXCR4 were 0.52 times (*P* < 0.05) and 0.78 times (*P* < 0.01) lower, respectively, after adding EG00229 for 10 h (Figure 7C).

The expression levels of PI3K, p-AKT, and p-mTOR proteins were all significantly decreased in A549-RR cells after adding EG00229 for 10 h, while AKT and mTOR were not significantly altered (Figure 7B). The mRNA expression level of PI3K was 0.22 times lower (*P* < 0.05), AKT was 0.73 times lower (*P* < 0.01) and mTOR was 0.87 times lower (*P* < 0.05) in A549-RR cells after adding EG00229 for 10 h (Figure 7C). In H1299-RR cells, the protein expression levels of PI3K, mTOR, and p-mTOR had all decreased, while AKT and p-AKT were not significantly altered (Figure 7B). The mRNA expression levels of PI3K, AKT, and mTOR decreased by 0.41, 0.49, and 0.73 times, respectively, after adding EG00229 for 10 h (all *P* < 0.01, Figure 7C).

## Discussion

In this study, we successfully constructed two models of radiation resistance in lung adenocarcinoma cells and demonstrated that EG00229 contributes to reversing the radiation resistance of lung adenocarcinoma cells.

In this study, we used an experimental method developed in our laboratory to construct A549-RR and H1299-RR cell models through multiple exposures to high doses of X-ray irradiation. There were some discrepancies in the cell cycle distribution of A549-RR and H1299-RR cells. To the best of our knowledge, A549 cells have KRAS point mutations and H1299 cells are naturally p53 deficient. Only cells with wild-type p53 can present with shortening of the G1 phase. Therefore, the difference in genetic backgrounds may be the reason for the difference in cell cycle distribution between A549-RR and H1299-RR cells. Further results indicated that radiation resistance was related to the upregulation of NRP1. These results are consistent with those reported by Zachary et al. 17. Therefore, we speculated that the mechanism of radiation resistance may be due to the altered expression of proteins in downstream pathways and the altered cell cycle processes caused by the increase in NRP1 expression.

Our results showed that the expression levels of EMT-related markers, including N-cadherin, β-catenin, vimentin, and α-SMA, were significantly increased in cells with radiation resistance. N-cadherin is a member of the calcium-dependent adhesion molecule family of classical cadherins that directly mediates homotypic and heterotypic cell-cell adhesion 18, 19. Vimentin is a cytoskeletal structural protein that is restricted to the cytosolic portion of cells 20, while α-SMA is a protein marker of mesenchymal cells 21. β-catenin is an integral structural component of cadherin-based adherens junctions and the key nuclear effector of canonical Wnt signaling in the nucleus 22. During EMT, epithelial cells undergo morphological changes from a cobblestone phenotype to an elongated fibroblastic phenotype, which is a biological process that plays an important role in embryonic development, cancer progression, and various fibrotic diseases and is strongly implicated in tumor cell invasion and metastasis 23. Upregulated NRP1 can induce the EMT of cells with radiation resistance.

We further investigated the mechanisms of radiation resistance by measuring the expression levels of certain proteins involved in tumor proliferation- and metastasis-related pathways (IL-6/STAT3, SDF-1/CXCR4, and PI3K/AKT/mTOR). The IL-6/STAT3 pathway is related to cell proliferation 24, while the SDF-1/CXCR4 and PI3K/AKT/mTOR pathways are related to metastasis. Our results showed that the expression levels of PI3K, p-AKT, and p-mTOR increased in cells with radiation resistance, indicating that upregulated NRP1 induced the activation of the PI3K/AKT/mTOR pathway, which can cause EMT in cells 25. Furthermore, we found that the expression levels of IL-6, STAT3, SDF-1, and CXCR4 were increased. STAT3 regulates a number of tumorigenesis-related pathways, such as cell cycle progression, apoptosis, tumor angiogenesis, invasion and metastasis, and tumor cell evasion of the immune system 26, 27. The increase in CXCR4 triggers actin polymerization, pseudopodia formation, and induces the targeted migration and invasion of tumor cells. SDF-1 is the only known ligand of CXCR4, and the activation of the SDF-1/CXCR4 signaling pathway is one of the key features of cell migration 28-30. Our results suggested that NRP1 can enhance the ability of NSCLC cells to metastasize and proliferate through regulation of the IL-6/STAT3 and SDF-1/CXCR4 signaling pathways.

After the addition of EG00229, which inhibits NRP1, the expression of NRP1 and VEGF-165 was decreased and the binding capacity of NRP1 and VEGF-165 was weakened. This indicates that EG00229 can decrease the expression of NRP1 by inhibiting the binding of NRP1 and VEGF-165. The expression of EMT-related proteins (N-cadherin, β-catenin, vimentin, and α-SMA) and proteins in the PI3K/AKT/mTOR pathway (PI3K, p-AKT, and mTOR) were reduced, indicating that the inhibition of NRP1 by EG00229 can inhibit the activation of the PI3K/Akt/mTOR pathway, weaken cell proliferation, and affect cell cycle progression. Similarly, we found that EG0029 significantly inhibited the activation of the SDF-1/CXCR4 and IL-6/STAT3 pathways, decreased the proliferation and migration of NSCLC cells, and further reduced the radiation resistance of NSCLC cells.

However, several limitations to this study should be considered. Initially, surgically resected radiotherapy-resistant human lung cancer samples were obtained from patients who had undergone radiation treatment before surgery. Adenocarcinoma has poor sensitivity to radiation and is easy to hematogenous metastasis, the current clinical treatment of lung cancer is mostly surgery, so surgically resected radiotherapy-resistant human lung cancer samples could not be obtained. Next, The radiation resistance model was established by fractionated irradiation, modeled after fractionated radiotherapy. The radiation resistance model could be established in cancer cells rather than human body or in human lung adenocarcinoma samples surgically removed. Finally, we only investigate the mechanistic role of NRP1 in the radiation resistance of non-small cell lung cancer cells, other tumors will be explored in future studies.

In summary, we successfully constructed two models of radiation resistance in lung adenocarcinoma cells using the experimental method developed in our lab and demonstrated new evidence for the role of NRP1 in the mechanism of radiation resistance in NSCLC cells. Because EG00229 contributes to reversing the radiation resistance of NSCLC cells, these findings could provide a new theoretical and experimental basis to improve the efficacy of lung cancer radiotherapy.

## Supplementary Material

Supplementary table S1.

## Figures and Tables

**Table 1:** The survival curve fitting results of single click multi target model

Group	D0 (Gy)	n	Dq (Gy)	SF_2_	R^2^
A549	2.272	2.005	1.581	0.612	0.963
A549-RR	1.503	25.260	4.854	0.952	0.972
H1299	1.931	3.188	2.239	0.706	0.967
H1299-RR	0.934	98.150	4.286	0.893	0.984

SF_2_ is survival fraction when irradiation dose is 2 Gy, D0 is the average lethal dose, Dq is quasi-field dose, R^2^ is coefficient of determination, and N is the extrapolation number.

## Abbreviations

NRP1: Neuropilin 1
NRPs: Neuropilins
NSCLC: non-small cell lung cancer
EMT: epithelial-mesenchymal transition
VEGF: Vascular endothelial growth factor
