# Supplementary material for: Effect of EG00229 on Radiation Resistance of Lung Adenocarcinoma Cells
Source: J Cancer. 2021 Aug 24;12(20):6105–17. doi: 10.7150/jca.56123 (PMC8425201; doi:10.7150/jca.56123)
Supplement: Supplementary file 1 — Supplementary table S1. [file jcav12p6105s1.pdf]

	Forward	Reverse
NRP1	5'-CAGGTGATGACTTCCAGCTCA-3'	5'-CCCAGTGGCAGAAGGTCTTG-3'
GAPDH	5'-GAAGGTGAAGGTCGGAGTC-3'	5'-GAGATGGTGATGGGATTTC-3'
N-cadherin	5'-AGCGCAGTCTTACCGAAGG-3'	5'-TCGCTGCTTTCATACTGAACTTT-3'
Vimentin	5'-TTTCTCCACGCCTCCAGTT-3'	5'-ATGTCTTCGGCCAGGTTGT-3'
β-catenin	5'-GGCAGGTCATCACCATCGG-3'	5'-CGTGTTGGCGTAGAGGTCTTT-3'
α-SMA	5'-GTGCTGTCCCTCTATGCCTCTGG-3'	5'-GGCACGTTGTGAGTCACACCATC-3'
IL-6	5'-CGGACAGCTTGAACAGAATGT-3'	5'-ACCATCCCACTCACACCTCA-3'
STAT3	5'-CAGCAGCTTGACACACGGTA-3'	5'-AAACACCAAAGTGGCATGTGA-3'
SDF-1	5'-TATTACTGGGACTGTGCTCAGAGA-3'	5'-TGATCAGGCTACAGAAATGAGAAG-3'
CXCR4	5'-TGTCATCT ACACAGTCAACCTC-3'	5'-CAACATAGACCACCTTTTCAGC-3'
PI3K	5′-CATCACTTCCTCCTGCTCTAT-3'	5'-GCCAGGGACACCTCCATCTC-3'
AKT	5′-AGCGACGTGGCTATTGTGAAG-3'	5'-GCCATCATTCTTGAGGAGGAAGT-3'
m-TOR	5'-AGAAACTGCACGTCAGCACCA-3'	5'-CCATCCAGCCAGTCATCTTTG-3'
